# Vertebral CT attenuation outperforms standard clinical fracture risk prediction tools in detecting osteoporotic disease in lung cancer screening participants

**DOI:** 10.1259/bjr.20220992

**Published:** 2023-09-03

**Authors:** Nikita Patel, Katrina Dahl, Rachael O'Rourke, Anne Williamson, Mark D Chatfield, Kwun M Fong, Ian A Yang, Henry M Marshall

**Affiliations:** 1 University of Queensland Thoracic Research Centre, Queensland, Australia; 2 Thoracic Medicine Department, The Prince Charles Hospital, Queensland, Australia; 3 Medical Imaging Department, The Prince Charles Hospital, Queensland, Australia; 4 Faculty of Medicine, University of Queensland, Queensland, Australia

## Abstract

**Objectives:**

Compare accuracy of vertebral Hounsfield Unit (VHU) attenuation and FRAX and Garvan Fracture Risk Calculators in identifying low bone mineral density (BMD) and prevalent vertebral compression fractures (VF) in lung cancer screening (LCS) participants.

**Methods:**

Baseline CT scans from a single site of the International Lung Screen Trial were analysed. BMD was measured using VHU (of the most caudally imaged vertebra) and quantitative CT (QCT) (low BMD defined as <110 HU and <120 mg/cm^3,^ respectively). Prevalent VF were classified semi-quantitatively. 10-year FRAX and Garvan fracture risks were calculated using dual energy X-ray absorptiometry (DXA) femoral neck T-score where available. Discrimination was assessed by area under receiver-operating characteristic curves (AUC).

**Results:**

535 LCS participants were included; 41% had low VHU-BMD, 56% had low QCT-BMD and 10% had ≥1 VF with ≥25% vertebral height loss. VHU demonstrated 94% specificity and 70% sensitivity in identifying low QCT-BMD. VHU was superior to fracture risk tools in discriminating low QCT-BMD (AUC: VHU 0.94 *vs* FRAX 0.67, Garvan 0.64 [*p* < 0.05]). In 64 participants with recent DXA scans, VHU was superior to FRAX_T-score_ and Garvan_T-score_ in discriminating low QCT-BMD (AUC: VHU 0.99, FRAX_T-score_ 0.71, Garvan_T-score_ 0.71 [*p* < 0.05]). VHU was non-inferior to FRAX_T-score_ and Garvan_T-score_ in discriminating VF (AUC: VHU 0.65, FRAX_T-score_ 0.53, Garvan_T-score_ 0.61).

**Conclusions:**

VHU outperforms clinical risk calculators in detecting low BMD and discriminates prevalent VF equally well as risk calculators with T-scores, yet is significantly simpler to perform.

**Advances in knowledge:**

VHU measurement could aid osteoporosis assessment in high-risk smokers undergoing LCS.

## Introduction

Lung cancer is the leading cause of cancer-related mortality worldwide. Lung cancer screening (LCS) with low-dose chest computed tomography demonstrated mortality benefits in large, international trials.^1,2^ LCS can potentially detect other diseases that share smoking as an aetiology, impacting patient outcomes, cost-effectiveness and generalisability. Osteoporosis represents a possible target disease: LCS participants may harbour undiagnosed osteoporosis due to the risks of smoking history and older age.^3,4^ Osteoporosis is often occult but can be detected by CT assessment of vertebral bone mineral density (BMD) and vertebral fractures (VF), prompting early intervention.

Standard clinical osteoporosis diagnosis involves fracture risk assessment and BMD testing. This is commonly achieved using multivariable risk calculators, such as FRAX or Garvan. BMD assessment, using dual-energy X-ray absorptiometry (DXA) or quantitative CT (QCT) improves clinical risk prediction, but recommendations vary internationally.^5–11^ A presumptive diagnosis of osteoporosis can also be made in the presence of a non-traumatic spinal compression fracture.^5^

The WHO-preferred BMD test is DXA, due to wide availability and low radiation dose. Although QCT has superior sensitivity for interval BMD change and avoids confounding factors, like degenerative bone disease and aortic calcification, which may overestimate areal DXA-BMD,^12^ it requires specialised analysis software. QCT is also limited by radiation dose, although this becomes irrelevant if pre-existing CT images are analysed.^12^

Due to high disease burden and economic cost of osteoporosis,^13^ early detection and fracture prevention in high-risk individuals is cost-effective.^14,15^ Smoking history alone does not qualify individuals for subsidised BMD testing in Australia, UK and USA. LCS participants aged below 70 years, although at risk of osteoporosis, are thus potentially excluded from BMD testing unless they have other significant risk factors or, in USA, UK and parts of Europe, if their estimated fracture risk meets a pre-defined clinical risk threshold.^5,6,10,11^

Finding scalable alternatives to established BMD tests represents an important goal for patient care, especially if incorporation into population-wide initiatives such as LCS can add value.

Hounsfield Unit-attenuation of the vertebrae (VHU) is a proxy BMD measure. VHU has been studied in CT performed for numerous clinical indications^16–18^ and demonstrates high sensitivity for DXA-defined osteoporosis.^19^ Low VHU is also associated with all-cause mortality in LCS participants.^3^ However, comparison against “standard-of-care” clinical fracture risk tools (with and without BMD T-scores) is necessary to support VHU as a screening tool in LCS.

To address this gap, we aimed to 1) compare VHU to FRAX and Garvan tools in discrimination of low QCT-BMD 2) compare VHU, QCT, FRAX and Garvan tools in discriminating prevalent VF and 3) assess VHU reproducibility in a LCS cohort.

## Methods

### Study design

This was a substudy of participants from a single site of the International Lung Cancer Screening Trial (ILST).^20^ ILST recruited healthy asymptomatic males and females, aged 55–80 years, at risk of lung cancer who met USPSTF eligibility criteria (current or former smokers who quit smoking ≤15 years ago, ≥30 pack-year history) and/or had an estimated PLCOm2012 lung cancer risk ≥1.51%) through media advertisements and press releases.^20–22^ Osteoporosis-relevant and other medical and demographic variables were prospectively collected at baseline. Baseline screening CT scans were analysed at the per-vertebra and per-participant level. Ethical approval was obtained through Hospital Human Research Ethics Committee. Participants provided written informed consent.

### CT acquisition

Participants underwent CT scans between 2017 and 2019, following a standard low-dose protocol (GE Healthcare Revolution^TM^ 256-detector row CT scanner, 120kV, 40-50mA, 1:0 pitch, 1.8 mSv mean effective dose recorded by a dose-tracking system). Reconstructed images (standard soft-tissue kernel with ≤1 mm thickness and spacing) were stored in the hospital picture archiving and communication system (PACS).

One observer assessed BMD and VF from all scans following training and assessment by a consultant radiologist and blinded validation against a subset of scans from a prior LCS study.^23^ Blinded QCT and VHU assessments were performed at least 2 weeks apart. The observer repeated blinded VHU assessments on 100 randomly selected scans after 4–6 months. Randomisation was performed in *Microsoft Excel*, using RAND function to assign a value, order then select every fifth scan.

### Hounsfield unit density assessment (VHU)

**Figure 1.**
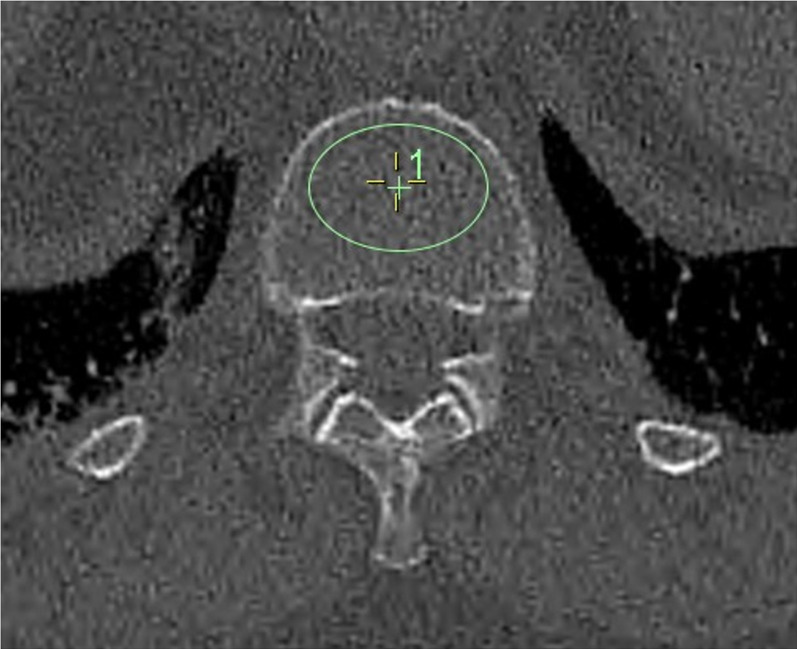
Example of region of interest (ROI) placement within the vertebral body for measurement of VHU (V1 shown in image).

VHU was measured following a published technique using standard *InteleViewer^TM^* software (Intelerad Medical Systems Inc., Montreal, QC, Canada).^19,24,25^ Reconstructed axial images were viewed in a bone window (W:2500 L:480, compression 4:1). Mean attenuation (Hounsfield units) was measured from a single 2D elliptical region-of-interest (ROI) placed in the anterosuperior portion of the L1 body, avoiding vertebral cortex, traversing veins and focal bony lesions (Figure 1). If L1 was partially imaged or fractured, the next superior, morphologically normal vertebra was measured. Low VHU-BMD (osteoporosis and osteopenia) was *a priori* defined as <110 HU.^24^

### Quantitative CT density assessment

Asynchronous QCT was performed using *Mindways QCT Pro^TM^* (Austin, TX, USA). Mean and individual volumetric BMD of imaged vertebrae T11-L1 were measured from semi-automated ROIs (9 mm vertical depth and similar axial area to VHU) placed in the upper half of each vertebral body. Mean T11-L1 QCT-BMD thresholds were defined using standard criteria: low<120 mg/cm^3^ (osteoporosis<80 mg/cm^3^; osteopenia 80–120 mg/cm^3^), normal ≥120 mg/cm^3^.^26^ Six-month quality control monitor studies were performed as per manufacturer guidelines.

### Vertebral fracture assessment

VF were assessed across all imaged vertebra from reconstructed mid-sagittal views. The semiquantitative Genant method was used, grading VF by proportional height loss: Grade 1 (mild), 20–25%; Grade 2 (moderate), 25–40%; Grade 3 (severe), >40%.^27^ Unequivocal VF (Grade 2–3) were deemed clinically significant. Sensitivity analysis including all VF grades^1–3^ was also performed.

### Clinical osteoporotic fracture risk assessment

Participant 10-year risk of hip or major osteoporotic fracture (hip, spine, distal forearm, or proximal humerus) was estimated using FRAX (Australia-specific version) and Garvan calculators excluding DXA T-score, using self-reported clinical variables.^28,29^ For participants with DXA studies ≤ 2 years prior to baseline CT scan, FRAX and Garvan risk were also calculated including femoral neck T-score (FRAX_T-score_ & Garvan_T-score_). As per treatment initiation thresholds, 10 year FRAX or Garvan hip fracture risk >3% or major osteoporotic fracture risk >20%, was deemed “high-risk”.^5,15^ Quality control of questionnaire data was performed by a physician. Missing or equivocal responses were clarified directly with participants by telephone. Reported fracture history was confirmed (in preferential order) against historical imaging, medical reports or detailed participant history. Fractures occurring from standing height or below, or due to circumstances that would not cause fracture in a healthy adult, were considered “low-trauma”.^10,28^ Risk factors unknown to participants were presumed absent. Participants with incomplete clinical data following quality control were excluded.

### Analysis

Statistical analysis was performed using *RStudio* (V1.3.1073, Boston, MA, USA) and Stata software (V17, StataCorp LLC, College Station, TX, USA). Means were compared using student’s t-test. Proportions were compared using chi-squared test. *p*-values < 0.05 distinguished statistical significance. Agreement between BMD methods was assessed using regression-based Bland-Altman analysis.^21^ Correlation was assessed using Pearson’s coefficient (*R*). Discrimination of methods (VHU, mean QCT-BMD, FRAX and Garvan 10 year risk of major osteoporotic fracture with and without DXA T-score) was assessed using area under receiver-operating characteristic curves (AUC). Optimal VHU threshold for discriminating low QCT-BMD was determined using Youden index. Associations between key variables and prevalent VF were modelled using logistic regression.

Intra- and inter-observer reliability of VHU was measured using intra-class correlation coefficient (ICC) and intra-observer reliability also using root-mean-square-error (RMSE). Inter-observer reliability was measured from blinded VHU measurements performed in a retrospective training data sample (*n* = 29) by clinicians of differing experience (resident physician, pulmonologist, radiologist and a radiology trainee).

### Sample size

A projected sample of ≥500 participants aimed to maximise incident fracture yield over the prospective 5-year trial period (estimated from previous Australian population-based cohort studies) in order to accurately assess these outcomes at trial completion.^30^

## Results

### Participants

**Figure 2.**
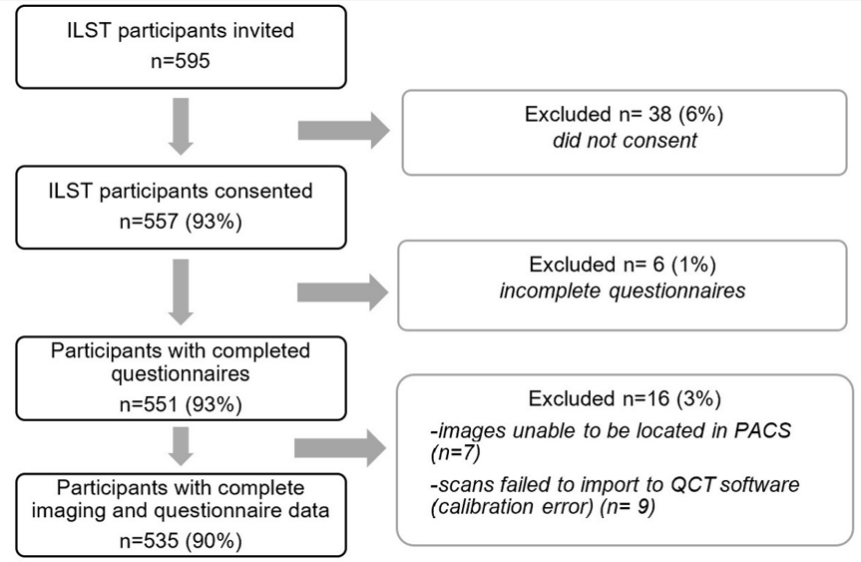
STARD Diagram describing flow of participants through study. ILST = International Lung Screening Trial. QCT = Quantitative CT. PACS = picture archiving and communication system.

Study flow is shown in Figure 2. Thirty-eight of 595 ILST participants did not provide informed consent; six had missing clinical risk information; 16 had technical issues with CT analysis (seven scans could not be retrieved from PACS for BMD analysis and nine scans encountered a calibration module error during QCT analysis).

535 included participants had complete CT and clinical data (median age 64 [IQR = 11] years; 315 [59%] male). 246 (46%) participants were active smokers (Table 1). Excluded participants were younger (median age 62 [IQR = 12] years), predominantly male (73%, *p* = 0.042) and somewhat more likely active smokers (58%; *p* = 0.093).

**Table 1.** Baseline characteristics of participants by highest prevalent vertebral fracture grade

Characteristic	No fracture (Grade 0) (*n* = 348)	Mild fracture (Grade 1) (*n* = 132)	Moderate or severe fracture (Grade two or 3) (*n* = 55)	Total (*n* = 535)
Male gender (n(%))	190 (55)	89 (67)	37 (67)	316 (59)
BMI (mean (SD)), kg/m^2^	28.5 (5)	29.2 (5)	27.4 (4)	28.6 (5)
Age (median (IQR)), years	64 (11)	65.0 (9)	67 (12)	64.8 (6)
Current smoker (n(%))	157 (45)	66 (50)	24 (44)	247 (46)
High fracture risk score^a^ (n(%))	109 (31)	42 (32)	31 (56)	182 (34)
High FRAX only	56 (16)	18 (14)	15 (27)	89 (17)
High Garvan only	88 (25)	37 (28)	29 (53)	154 (29)
High FRAX and Garvan	35 (10)	13 (10)	13 (24)	61 (11)
VHU (mean(SD)), HU	125 (32)	114 (34)	102 (29)	120 (33)
QCT (mean(SD)), mg/cm^3^	126 (30)	115 (33)	102 (30)	121 (32)
Low VHU BMD (<110 HU) (n(%))	120 (34)	66 (50)	34 (62)	220 (41)
Low QCT BMD (<120 mg/cm^3^) (n(%))	166 (48)	88 (67)	45 (81)	299 (56)
Osteopenia (80–120 mg/cm^3^)	150 (43)	78 (59)	34 (62)	262 (49)
Osteoporosis (<80 mg/cm^3^)	16 (5)	10 (8)	11 (20)	37 (7)

BMD, bone mineral density; QCT, quantitative CT; VHU, Vertebral Hounsfield unit CT-attenuation.

a FRAX or Garvan 10 year risk≥3% of hip fracture or≥20% of any major osteoporotic fracture, without T-score.

### Vertebral BMD assessments

To align with standard spinal DXA and QCT, which measure BMD from the lumbar spine, we assessed BMD based on the most caudal vertebra available. L1 was imaged in 277 participants, of which five had a fracture and one was sclerotic. VHU was thus measured from L1 (*n* = 271), T12 (*n* = 199) T11 (*n* = 55) and T10 (*n* = 10). Mean L1 attenuation (116 ± 31 HU) was significantly lower than mean T12 attenuation (122 ± 32 HU, *p* = 0.047).

QCT-BMD was measured at L1 (*n* = 271), T12 (*n* = 480), T11 (*n* = 519) and T10 (*n* = 7). Where vertebrae T11-L1 were measured simultaneously (*n* = 265), bone density showed a cranio-caudal gradient; mean individual QCT-BMD was lowest at L1 (111 ± 31 mg/cm^3^), compared to T12 (116 ± 31 mg/cm^3^) and T11 (123 ± 30 mg/cm^3^). QCT-BMD of each individual vertebra correlated strongly with mean T11-L1 BMD (*r* > 0.98). However, L1 BMD was significantly lower than mean combined T11-L1 BMD (117 ± 30 mg/cm^3^, *p* = 0.035). Mean T11-L1 BMD was used as the reference standard for analysis.

### Prevalence of low BMD

220 (41% [95% CI:37–45%]) participants had low VHU. 299 (56% [95% CI: 52–60%]) participants had low mean QCT-BMD (male to female: 1.23:1), of whom 262 (49% [95% CI: 45–53%]) had osteopenia and 37 (7% [95% CI: 5–9%]) had osteoporosis (Table 1).

139 (26%) participants had prior DXA scans. Reports for 110 participants were located (dated 1997 to 2018); 17 (15%) and 72 (65%) participants met osteoporosis and osteopenia criteria, respectively.^7^ In 64 participants with DXA within prior 2 years, QCT identified DXA-defined osteoporosis with sensitivity 0.29 and specificity 0.94, and identified DXA-defined osteoporosis or osteopenia with sensitivity 0.80 and specificity 0.5. VHU identified DXA-osteoporosis or osteopenia with sensitivity 0.70 and specificity 0.64.

Of 396 (74%) participants without prior BMD testing, 202 (49%) met Australian Medicare eligibility for subsided BMD testing based on age and risk factors.^5^ 180/396 (45%) participants had QCT-osteopenia and 22/396 (6%) had QCT-osteoporosis.

### Prevalence of high osteoporotic fracture risk

182 (34% [95% CI: 30–38%]) participants had high FRAX and/or Garvan risk for hip or major osteoporotic fracture (Table 1). This included 28/37 participants with QCT-osteoporosis (76% [95%CI: 60–87%]), 97/262 participants with QCT-osteopenia (37% [95%CI: 31–43%]) and 103/220 participants with low VHU (47% [95%CI: 40–53%]).

### Prevalence of vertebral fractures

Genant Grades 2–3 VF were present in 55 (10% [95% CI: 8–13%]) participants (male to female: 2:1)(Table 1). 187 participants had VF of any grade: Grade 1 was the highest grade in 132 (71% [95% CI: 63–77%]) participants, Grade 2 was highest in 42 (22% [95% CI: 17–29%]) participants and Grade 3 was highest in 13 (7% [95% CI: 4–12%]) participants.

In total, 323 VF were detected across all imaged vertebrae: 249 VF were Grade 1, 59 were Grade 2 and 15 were Grade 3. Significant VF were most commonly detected at T7 (16%) and T8 (16%) followed by T9 (12%), T12 (12%) and L1 (12%).

Mean VHU was lower in participants with prevalent VF (*p* < 0.001). Each 10HU increase in VHU was associated with a 21% decreased odds of prevalent VF (OR:0.79 [95%CI: 0.71–0.88], *p* < 0.001) (Supplementary Table 1).

Supplementary Material 1.

In multivariable analysis, male gender was associated with a 92% increased odds of a prevalent VF (OR:1.92, [95%CI: 1.04–3.67]) (Supplementary Table 2). Age, BMI and active smoking did not reach statistical significance (Supplementary Table 2). Results were similar when all VF grades were included (Supplementary Table 3 and 4).

### Comparison of methods in classifying low BMD

**Figure 3.**
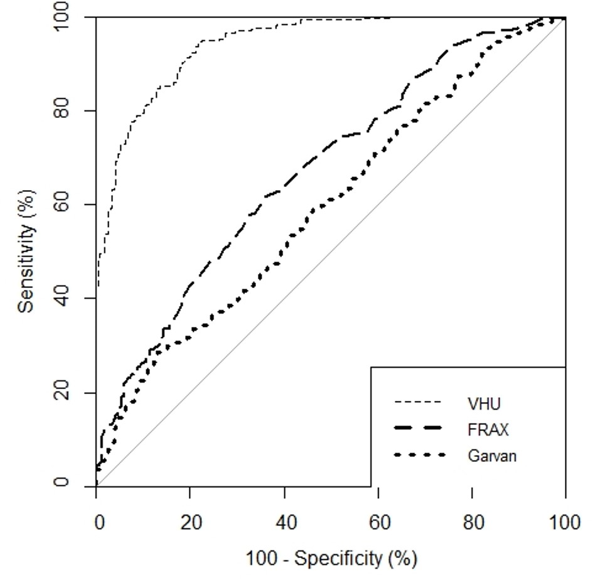
Receiver-operating characteristic curves showing vertebra Hounsfield Unit attenuation (VHU) compared to clinical risk scores (FRAX and Garvan) in discriminating low quantitative CT-defined bone mineral density.

VHU accurately discriminated individuals with low mean QCT-BMD (AUC 0.94 [95%CI: 0.93–0.96])(Table 2, Figure 3). At a threshold of 110HU, VHU had specificity 0.95 (95%CI: 0.92–0.97), sensitivity 0.70 (95%CI: 0.65–0.75), positive predictive value 0.95 (95%CI: 0.91–0.97) and negative predictive value 0.71 (95%CI: 0.66–0.76) in detecting low QCT-BMD. We performed sensitivity analysis of L1 and T12 vertebral levels separately. Discrimination of low mean QCT-BMD was not significantly different between L1 VHU (AUC 0.96 [95%CI: 0.93–0.98]) and T12 VHU (AUC 0.91 [95%CI: 0.88–0.95], *p* = 0.06) (Supplementary Table 5).

**Table 2.** AUC for variables in classifying low BMD and prevalent vertebral fracture in the entire study cohort (*n* = 535) and in a subset of individuals with DXA T-scores (*n* = 64)

	Variable	Discrimination of low BMD (QCT<120 mg/cm^3^)		Discrimination of prevalent VF (moderate or severe only)		Discrimination of prevalent VF (any grade)	
		AUC	95% CI	AUC	95% CI	AUC	95% CI
All participants (*n* = 535)	QCT	NA		0.72	0.64–0.79	0.66	0.61–0.71
	VHU	0.94	0.93–0.96	0.68	0.61–0.75	0.64	0.59–0.69
	FRAX^a^	0.67	0.63–0.72	0.57	0.49–0.66	0.51	0.46–0.56
	Garvan^a^	0.60	0.55–0.64	0.55	0.46–0.65	0.50	0.45–0.56
Participants with DXA T-score (*n* = 64)	QCT	NA		0.68	0.49–0.86	0.67	0.53–0.81
	VHU	0.99	0.97–1.00	0.65	0.44–0.87	0.60	0.45–0.74
	FRAX^a^	0.73	0.60–0.87	0.42	0.21–0.62	0.43	0.28–0.57
	FRAX_T-score_^a^	0.71	0.56–0.87	0.53	0.33–0.73	0.52	0.37–0.66
	Garvan^a^	0.74	0.62–0.87	0.55	0.35–0.75	0.51	0.36–0.86
	Garvan_T-score_^a^	0.71	0.57–0.86	0.61	0.41–0.80	0.61	0.46–0.75

AUC, area under receiver-operating curve; BMD, bone mineral density; QCT, quantitative CT (reference standard); VF, vertebral fracture; VHU, Vertebral Hounsfield unit.

a FRAX or Garvan 10-year risk of any major osteoporotic fracture, calculated without T-score unless specified.

VHU outperformed clinical risk calculators in discriminating low QCT-BMD (AUC: FRAX 0.67 [95%CI: 0.63–0.72] and Garvan 0.60 [95%CI: 0.55–0.64]) (Figure 3). In 64 participants with recent DXA, VHU outperformed FRAX and Garvan risk tools (both with and without T-score) in discriminating low QCT-BMD (AUC: VHU 0.99 [95%CI:0.97–1]; FRAX_T-score_ 0.71 [95%CI:0.56–0.87]; FRAX 0.73 [95%CI:0.60–0.87]; Garvan_T-score_ 0.71 [95%CI: 0.57–0.86]; Garvan 0.74 [95%CI:0.62–0.87]) (Table 2).

Optimal VHU threshold to classify QCT-osteoporosis or osteopenia was 126HU (specificity 0.78, sensitivity 0.94). Optimal threshold to classify QCT-osteoporosis was 95HU (specificity 0.84, sensitivity 1.00).

### Comparison of methods in classifying prevalent fracture

**Figure 4.**
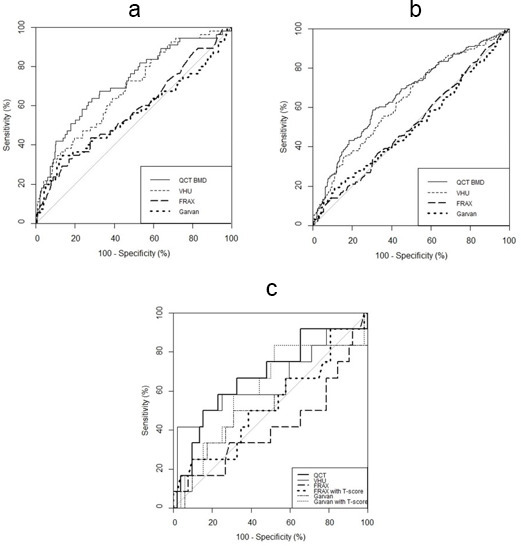
Receiver-operating characteristic curves showing: (**a**) Bone mineral density measurement methods (quantitative CT [QCT] and vertebra Hounsfield Unit attenuation [VHU]) compared to clinical risk scores (FRAX and Garvan) in discriminating prevalent moderate to severe vertebral fracture (Grade 2–3). (**b**) Bone mineral density measurement methods (QCT and VHU) compared to clinical risk scores in discriminating any prevalent vertebral fracture (Grade 1–3). (**c**) VHU compared to FRAX (with and without T-score) in discriminating prevalent moderate to severe vertebral fracture (Grade 2–3).

VHU and QCT showed moderate discrimination of individuals with prevalent VF and were superior to clinical risk tools without T-score, AUC (95% CI) VHU, QCT, FRAX and Garvan 0.68 (0.61–0.75), 0.72 (0.64–0.79), 0.57 (0.49–0.66) and 0.55 (0.46–0.65), respectively, and similar in sensitivity analysis (Table 2, Figure 4a and Figure 4b).

In participants with previous DXA, VHU was non-inferior to FRAX and Garvan tools (both with or without T-score) in discrimination of prevalent VF: AUC (95% CI) VHU 0.65 (0.44–0.87); FRAX_T-score_ 0.53 (0.33–0.73); FRAX 0.42 (0.21–0.62); Garvan_T-score_ 0.61 (0.41–0.80); Garvan 0.55 (0.35–0.75).

### Correlation and agreement between methods

**Figure 5.**
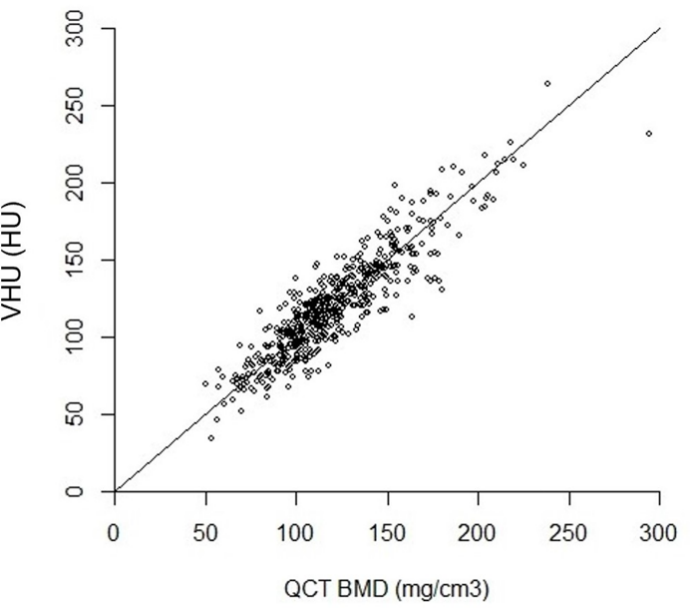
Scatter plot of quantitative CT (QCT) against vertebra Hounsfield Unit attenuation (VHU) measurements, showing high correlation (*R* = 0.90).

VHU correlated highly with QCT-BMD (*R* = 0.90, RMSE 14.1) (Figure 5). In Bland-Altman analysis, mean difference between VHU and QCT values for an individual was −0.73. Regression between means and differences showed little difference on average throughout the measurement range (Supplementary Figure 1). Smaller values rarely differed by more than 20 units, and larger values rarely differed by more than 40 units.

### Intra- and Inter-observer reliability

Intra-observer reliability between repeated VHU measurements was high with ICC 0.99 (95%CI: 0.98–0.99) and RMSE 4.1HU. Inter-observer reliability (*n* = 27 readings, one erroneous reading excluded) was excellent between four independent observers (ICC:0.93 [95%CI: 0.87–0.96]).

## Discussion

In line with prior studies, our high-risk LCS cohort harboured high prevalence of QCT-defined osteoporosis (7%) and osteopenia (49%), occult in 51% of participants. Using spinal DXA, the NELSON trial reported a higher osteoporosis prevalence of 27% but similar osteopenia prevalence of 44% (*n* = 302; 98 men).^25^ Another multi-centre study of LCS participants reported osteoporosis and osteopenia prevalence of 15 and 42%, respectively, using L1-L2 QCT (*n* = 34,153; 59% men).^31^ Our lower reported osteoporosis prevalence may arise from using higher vertebrae (T11 and T12) in QCT analysis.^32^

Vertebral fractures (VF) appeared highly prevalent in our cohort (35%), and in the NELSON cohort (39%).^3^ We note male gender was associated with increased odds (1.92) of prevalent VF, despite females generally experiencing a higher rate of BMD decline post-menopause. We suggest that osteoporosis risk assessment is perhaps more readily undertaken in females, leading to increased use of bone protection medication, however, we do not have data to confirm or refute this.

VHU performed well against reference-standard QCT. Previous studies similarly report high discrimination of VHU against QCT. A recent study of non-contrast spinal LDCT (*n* = 180) reported an AUC of 0.97.^17^ Another LCS study (*n* = 457) reported AUC of 0.96 on comparing thoracic and upper lumbar vertebral attenuation to QCT.^4^ AUC values appear comparatively lower when comparing VHU to DXA (0.74 to 0.79^19,25^), likely due to confounding factors affecting areal DXA assessment.^12^ A validated method like asynchronous QCT may seem favourable, however the practicality of image transfer, translation and analysis, is more time-consuming when compared to a simple 30 s VHU measurement. While it is not surprising that VHU performs well against QCT, as both are based on Hounsfield Unit measurement, VHU is not approved for use in bone density screening; however, our data support the notion that VHU presents a scalable alternative in a screening setting.

VHU threshold of 110HU was highly specific but only moderately sensitive in classifying low QCT-BMD. This threshold, previously reported to be 91% specific in detecting DXA-defined osteoporosis using contrast-enhanced and non-contrast abdominal CT scans, was suggested for lower risk populations to minimise false-positive results.^24^ However, optimal thresholds vary between CT methods and cohorts, limiting generalisability.^16^ In a high-risk LCS cohort, more sensitive VHU cut-offs of 126HU (osteoporosis and osteopenia) and 95HU (osteoporosis) may be more appropriate to limit false-negative results.

VHU closely estimates BMD, the risk factor most strongly associated with fracture.^28^ In the absence of DXA T-score, VHU outperformed FRAX and Garvan in discriminating participants with low QCT-BMD and prevalent VF. Whilst inclusion of T-score reportedly improves the predictive ability of clinical tools for incident fractures,^9^ we did not find significant improvement in prediction of prevalent VF, which itself is predictive of incident fracture.^28^ VHU was similar to both FRAX_T-score_ and Garvan_T-score_ in discriminating participants with VF (AUC 0.53 and 0.61, respectively). This finding is important; risk estimation using FRAX and Garvan algorithms requires additional clinical information (subject to recall bias) and DXA imaging or QCT analysis, while, in comparison, measuring VHU is rapid, simple and reproducible, using non-specialised software on existing low-dose CT images. We found high intra-observer ICC 0.99 and inter-observer ICC 0.93, obtained from a sample of clinicians with varying levels of radiological experience, supporting method accessibility. This is consistent with previous studies which reported excellent inter- and intra-observer reliability ICC 0.96 and 0.94–0.98, respectively.^18^ Our results support further exploration of VHU in incident fracture prediction. To address this, our cohort will be followed up for health and fracture outcomes over the 5-year ILST trial period.

Recent large studies in USA and Israel compared automated CT-derived bone metrics from chest and abdominal CT scans against FRAX scores algorithmically calculated from electronic health records (EHR) from general populations and colon cancer screenees. They found automated CT-derived bone metrics were comparable with FRAX scores in predicting incident fractures.^33,34^ In contrast, our study employed clinician-conducted VHU measurements and clinician-validated FRAX calculation, replicating standard clinical workflow and reducing bias attributed to missing data. Our findings are relevant to clinical practice in the absence of an EHR, applicable in any clinical setting. Aligning with the findings of these studies, our results add to the generalisability of using bone density metrics in low-dose chest CT scans for high-risk LCS participants.

This study has some limitations. Unlike dedicated spine QCT scans, lumbar vertebrae are not always imaged on LCS CT scans, and thus VHU was not consistently measured from the same level. The decreasing intervertebral BMD gradient from T11 to L1 may cause under-recognition of osteoporosis when measuring BMD in thoracic rather than lumbar vertebrae.^4,32^ Although we found minimal difference between T12 and L1 attenuation in low QCT-BMD discrimination (AUC 0.92 and 0.96 respectively), consistent attenuation measurement from a higher vertebral level could be considered in further studies with a view to establishing thresholds for low-BMD classification. Similarly, using the average QCT-BMD value across T11-L1 may underestimate osteoporosis compared to the standard L1-L4 average.^32^ VF occurring outside the imaged field were also potentially omitted, contributing to bias. However as osteoporotic VF most commonly occur in the thoracic spine or thoracolumbar junction, most VF are likely to be captured in this study.^35^

Although QCT was used as reference-standard, DXA is the preferred standard test. While comparison between QCT and DXA was not our primary focus, we reported low sensitivity for QCT (35%) in identifying participants with prior DXA-defined osteoporosis, and low specificity (41%) in identifying prior DXA-defined osteoporosis or osteopenia within a small sample. As QCT is more sensitive to low BMD than DXA, “false-positives” may represent osteoporotic individuals undetected by DXA. Conversely, low specificity of QCT for detecting low DXA-BMD could be explained by measurement of higher vertebrae for QCT compared to DXA.

Our targeted study population of LCS participants limits wider applicability of results to non-screening cohorts due to reported variability in diagnostic accuracy of VHU.^16^ The narrow age window of LCS participants also limits applicability to younger smokers. However, LCS is rapidly becoming standard practice in many countries, with 14.5 million screen-eligible individuals estimated in USA alone.^36^ With well-defined participant selection and LDCT protocols internationally, our results will have widespread generalisability to large LCS cohorts.

In summary, we conclude that VHU can more simply and accurately discriminate LCS participants with occult osteoporotic disease, compared to standard-of-care fracture risk tools, regardless of prior T-score estimates. Among these individuals, who may not routinely access formal bone density testing, opportunistic VHU assessment and routine reporting on screening scans will add value to LCS, facilitating early disease detection and prompt intervention.
