# Supplementary material for: Vertebral CT attenuation outperforms standard clinical fracture risk prediction tools in detecting osteoporotic disease in lung cancer screening participants
Source: Br J Radiol. 2023 Sep 3;96(1151):20220992. doi: 10.1259/bjr.20220992 (PMC10607405; doi:10.1259/bjr.20220992)

**Supplementary Material**

Table S1: Univariable logistic regression analyses of moderate/severe vertebral fracture (Grade 2 or 3)

| Variable | Category | OR | 95% CI | | p-value | Global p-value |
| --- | --- | --- | --- | --- | --- | --- |
| VHU (HU) | Per 10 units | 0.79 | 0.71 | 0.88 | <0.001 |  |
| QCT (mg/cm^3^) | Per 10 units | 0.76 | 0.67 | 0.85 | <0.001 |  |
| Age (years) | <65 | ref |  |  |  | 0.22 |
|  | 65-69 | 0.98 | 0.45 | 2.03 | 0.96 |  |
|  | ≥70 | 1.67 | 0.88 | 3.13 | 0.11 |  |
| Gender | Male | 1.49 | 0.84 | 2.8 | 0.18 |  |
| BMI (kg/m^2^) | <25 | ref |  |  |  |  |
|  | 25-<30 | 1.52 | 0.76 | 3.20 | 0.25 | 0.14 |
|  | 30-<35 | 1.09 | 0.48 | 2.53 | 0.83 |  |
|  | ≥35 | 0.17 | 0.01 | 0.91 | 0.10 |  |
| Current smoker | Yes | 0.90 | 0.51 | 1.57 | 0.71 |  |

BMD = bone mineral density. VHU = Vertebral Hounsfield unit CT-attenuation. OR = odds ratio. QCT = quantitative CT.

Table S2: Multivariable logistic regression analyses of moderate/severe vertebral fracture (Grade 2 or 3)

| Variable | Category | OR | 95% CI | | p-value |
| --- | --- | --- | --- | --- | --- |
| VHU (HU) | Per 10 units | 0.77 | 0.68 | 0.86 | <0.001 |
| Age (years) | <65 | Ref |  |  |  |
|  | 65-69 | 0.60 | 0.26 | 1.32 | 0.22 |
|  | ≥70 | 0.86 | 0.41 | 1.77 | 0.68 |
| Gender | Male | 1.92 | 1.04 | 3.67 | 0.043 |
| BMI (kg/m^2^) | <25 | Ref |  |  |  |
|  | 25-<30 | 1.77 | 0.84 | 3.90 | 0.14 |
|  | 30-<35 | 1.10 | 0.46 | 2.67 | 0.83 |
|  | ≥35 | 0.19 | 0.01 | 1.05 | 0.12 |
| Current smoker | Yes | 0.95 | 0.50 | 1.78 | 0.88 |

VHU = Vertebral Hounsfield unit CT-attenuation. OR = odds ratio

Table S3: Univariable logistic regression analyses of any grade vertebral fracture (Grade 1-3)

| Variable | Category | OR | 95% CI | | p-value | Wald p-value |
| --- | --- | --- | --- | --- | --- | --- |
| VHU (HU) | Per 10 units | 0.87 | 0.81 | 0.92 | <0.001 |  |
| QCT (mg/cm^3^) | Per 10 units | 0.85 | 0.79 | 0.90 | <0.001 |  |
| Age (years) | <65 | ref |  |  |  | 0.47 |
|  | 65-69 | 1.21 | 0.77 | 1.89 | 0.40 |  |
|  | ≥70 | 1.28 | 0.83 | 1.95 | 0.26 |  |
| Gender | Male | 1.68 | 1.16 | 2.44 | 0.01 |  |
| BMI (kg/m^2^) | <25 | ref |  |  |  | 0.54 |
|  | 25-<30 | 1.31 | 0.83 | 2.10 | 0.25 |  |
|  | 30-<35 | 1.36 | 0.81 | 2.28 | 0.24 |  |
|  | ≥35 | 1.01 | 0.51 | 1.96 | 0.97 |  |
| Current smoker | Yes | 1.10 | 0.77 | 1.58 | 0.58 |  |

VHU = Vertebral Hounsfield unit CT-attenuation. OR = odds ratio. QCT = quantitative CT.

Table S4. Multivariable logistic regression analyses of any grade vertebral fracture (Grade 1-3)

| Variable | Category | OR | 95% CI | | p-value |
| --- | --- | --- | --- | --- | --- |
| VHU (HU) | Per 10 units | 0.84 | 0.78 | 0.90 | <0.001 |
| Age (years) | <65 | ref |  |  |  |
|  | 65-69 | 0.96 | 0.59 | 1.54 | 0.86 |
|  | ≥70 | 0.88 | 0.54 | 1.42 | 0.60 |
| Gender | Male | 1.95 | 1.32 | 2.91 | <0.001 |
| BMI (kg/m^2^) | <25 | ref |  |  |  |
|  | 25-<30 | 1.52 | 0.93 | 2.51 | 0.10 |
|  | 30-<35 | 1.46 | 0.84 | 2.55 | 0.18 |
|  | ≥35 | 1.18 | 0.57 | 2.40 | 0.65 |
| Current smoker | Yes | 1.28 | 0.86 | 1.90 | 0.22 |

VHU = Vertebral Hounsfield unit CT-attenuation. OR = odds ratio.

Table S5. AUC for vertebral attenuation, separated by vertebral level, in classifying low QCT-BMD and prevalent vertebral fractures

| Vertebral level | Discrimination of low BMD (QCT<120mg/cm^3^) | | Discrimination of prevalent VF (moderate or severe only) | | Discrimination of prevalent VF (any grade) | |
| --- | --- | --- | --- | --- | --- | --- |
|  | AUC | 95%CI | AUC | 95%CI | AUC | 95%CI |
| T11 | 0.98* | 0.95-1.00 | 0.77 | 0.65-0.90 | 0.61 | 0.43-0.78 |
| T12 | 0.91* | 0.88-0.95 | 0.62 | 0.43-0.80 | 0.57^#^ | 0.48-0.65 |
| L1 | 0.96 | 0.93-0.98 | 0.69 | 0.59-0.79 | 0.69^#^ | 0.62-0.75 |
| *Statistically significant difference (p<0.05)  ^#^Statistically significant difference (p<0.05)  BMD = bone mineral density. VHU = Vertebral Hounsfield unit CT-attenuation. QCT = quantitative CT. VF = vertebral fracture | | | | | | |

Figure S1: Bland Altman plot showing differences against means of paired quantitative CT (QCT) against vertebral Hounsfield Unit attenuation (VHU) measurements. The regression line (middle grey line) shows negligible proportional bias (slope=0.03).


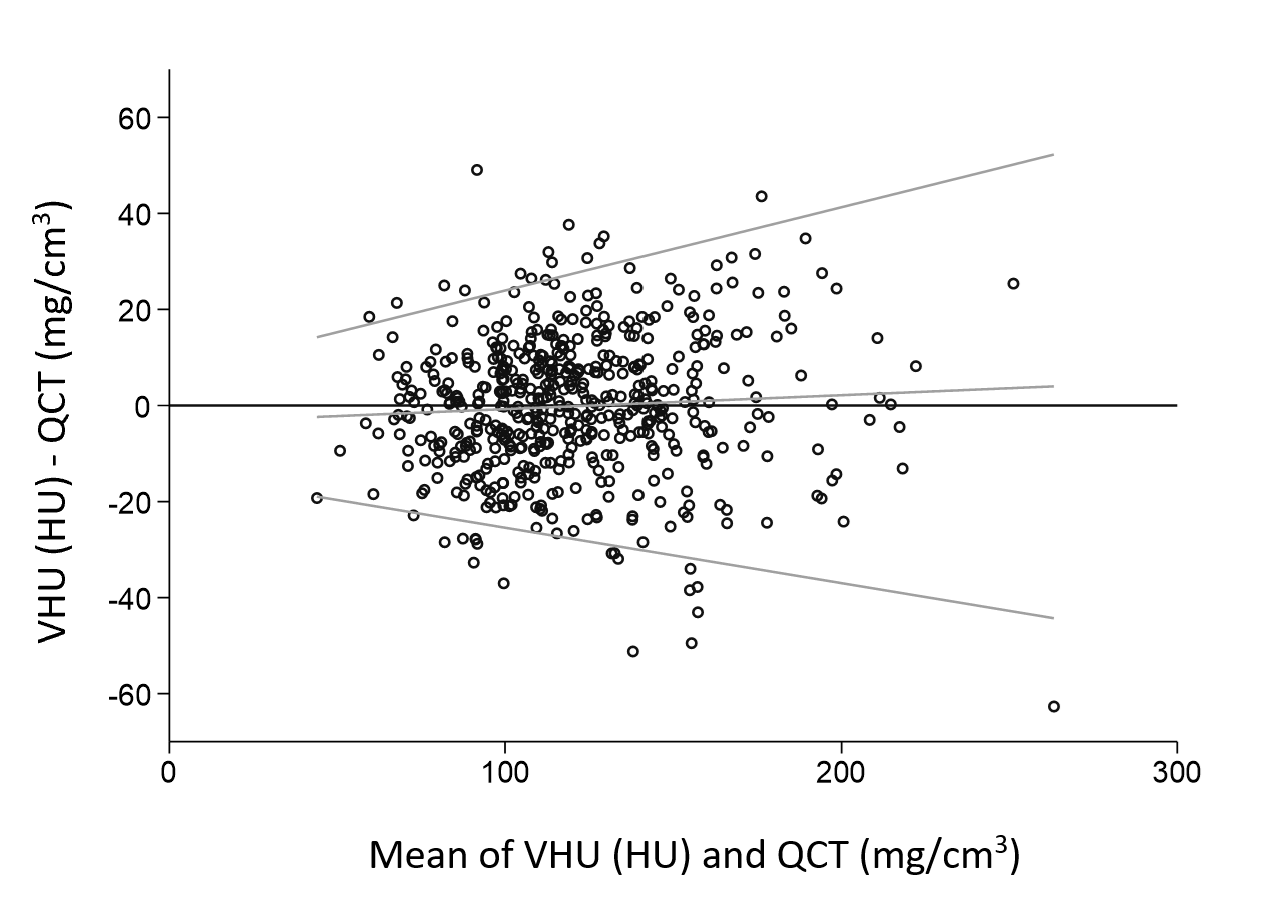

Supplement: Supplementary Material 1. [file bjr.20220992.suppl-01.docx]
